# Supplementary material for: Effects of the Expression of Random Sequence Clones on Growth and Transcriptome Regulation in Escherichia coli
Source: Genes (Basel). 2021 Dec 24;13(1):53. doi: 10.3390/genes13010053 (PMC8775113; doi:10.3390/genes13010053)
Supplement: Supplementary file 1 [file genes-13-00053-s001.zip › Bhave_Tautz_Suppl.pdf]

Supplementary files

Supplementary file S1

Expression vector and insert design. The IPTG inducible expression vector pFLAG-CTC was used to clone random sequence ORF inserts into the multiple cloning site (MCS) between the HindIII and SalI sites, as shown on top. The scheme of the full-length peptide product expressed upon induction is shown at the bottom.

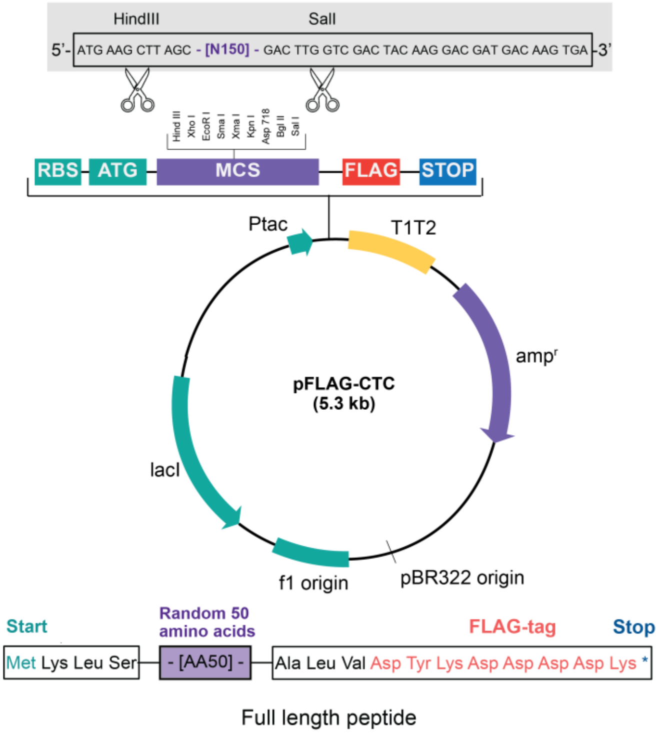

Supplementary file S2

Sequences of clones in this study: coding parts of the inserts are shown with their translation products. Constant parts derived from the vector sequences are shaded in grey.

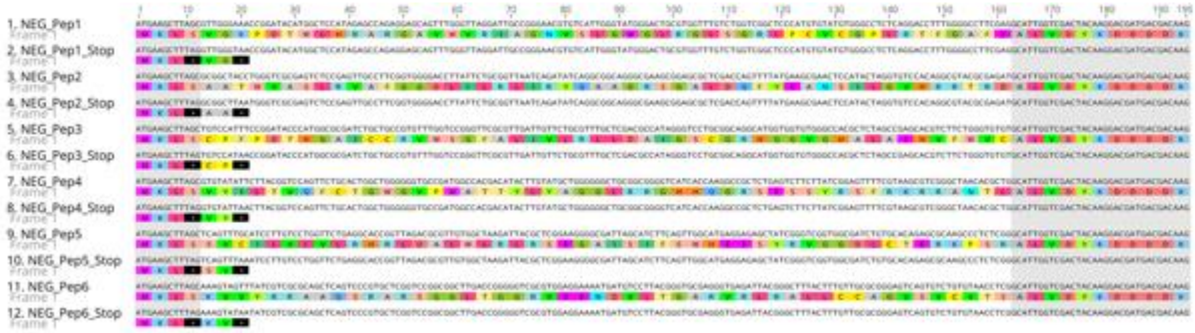

S2A: NEG clones and their STOP codon variants.

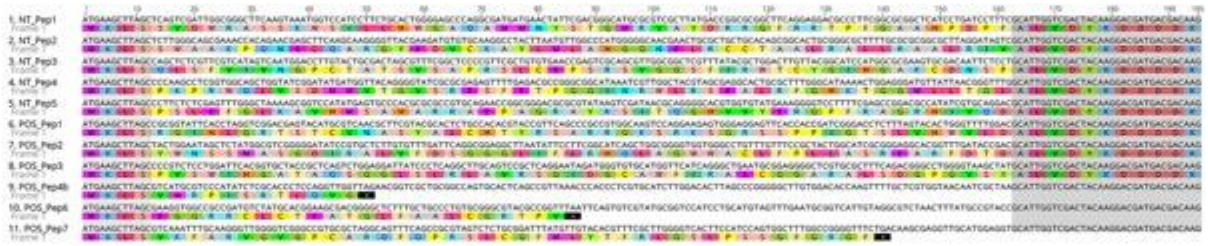

S2B: NT and POS clones.

Supplementary file S3

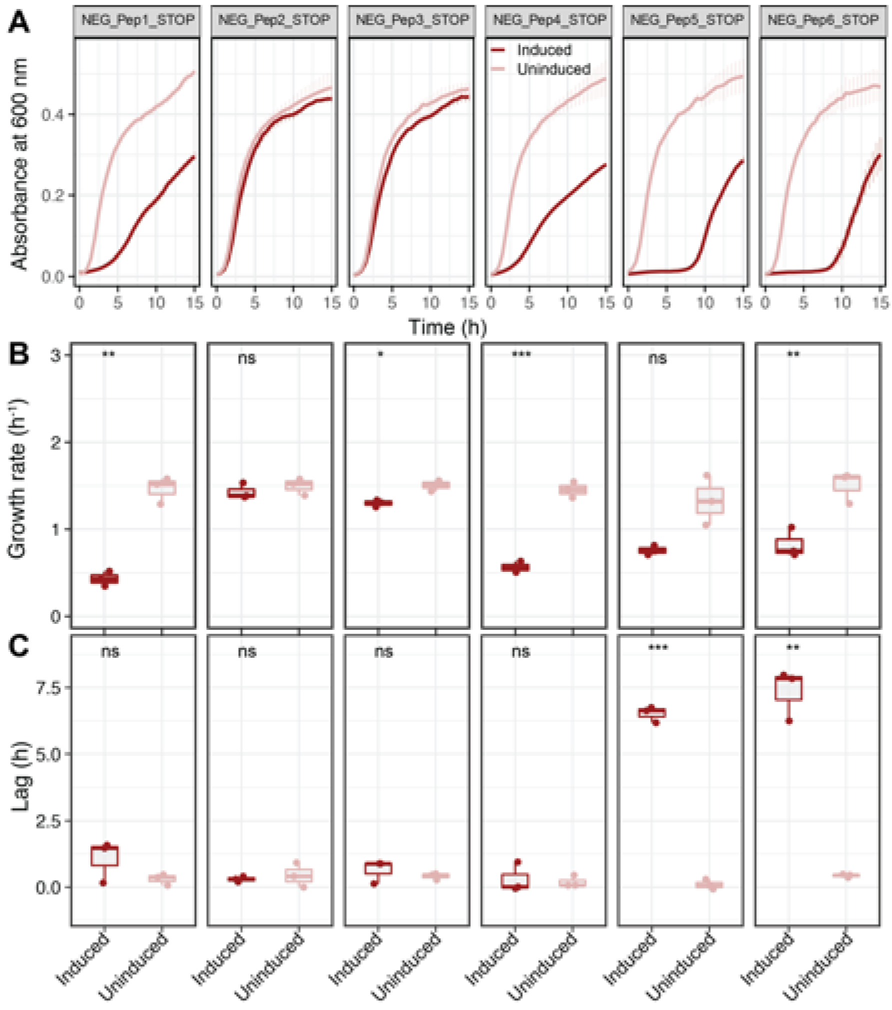

Growth rate comparisons for NEG peptide STOP clones. A) Growth curves without IPTG induction are depicted as light colours and with IPTG induction are in dark colours. OD600 was recorded every 10 min with at least five replicates for each clone and condition. Dots represent means and whiskers, which show the standard error of the mean (SEM) across replicates. B) Growth rate comparisons between the six clones after the lag. C) Lag time comparisons between the six NEG STOP clones, measured as the time from the start of the experiment until the start of the exponential growth. Student's t-test was performed with p-values as follows: *** = P < 0.001, ** = P < 0.01, * = P < 0.05, ns = P > 0.05.

Supplementary file S4

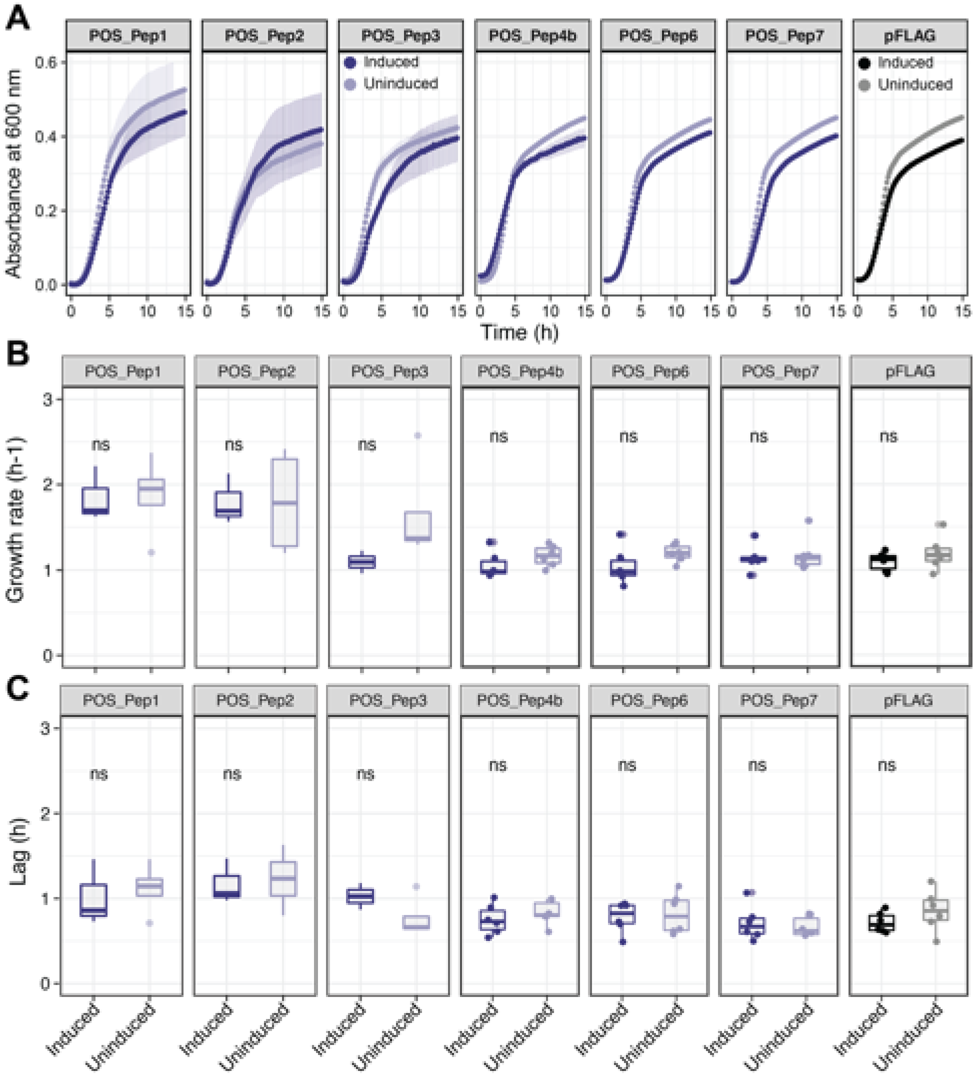

Growth rate comparisons for POS peptide clones compared to empty vector. A) Growth curves without IPTG induction are depicted as light colours and with IPTG induction are in dark colours. OD600 was recorded every 10 min with at least five replicates for each clone and condition. Dots represent means and whiskers, which show the standard error of the mean (SEM) across replicates. B) Growth rate comparisons between the six clones. C) Lag time comparisons between the six clones, measured as the time from the start of the experiment until the start of the exponential growth. Student's t-test was performed with p-values as follows: *** = P < 0.001, ** = P < 0.01, * = P < 0.05, ns = P > 0.05.

Supplementary file S5

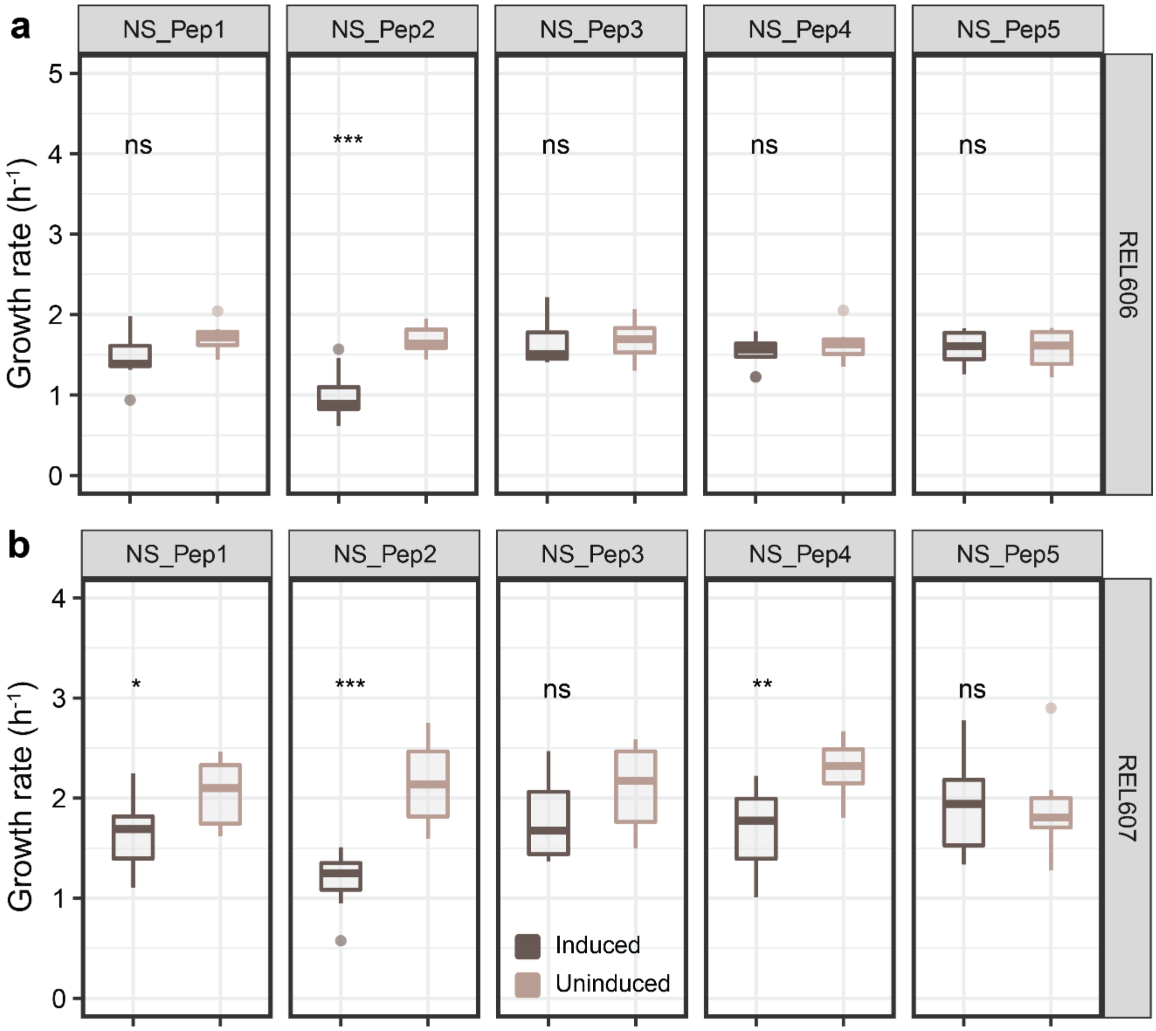

Growth rate comparisons for NS peptide clones in two different backgrounds. A) Growth rate comparisons between the five clones in REL606. B) Growth rate comparisons between the five clones in REL606.Student's t-test was performed with p-values as follows: *** = P < 0.001, ** = P < 0.01, * = P < 0.05, ns = P > 0.05.

Supplementary Table 1

Primer sequences used in this study

Name	Primer	Sequence
NEG_Pep1	Forward	ATGAAGCTTAGCGTTGGGAAACCGGATACATGGC
NEG_Pep1	Reverse	GTAGTCGACCAATGCCTCGAAGGCCCCAAAGGTC
NEG_Pep1_STOP	Forward	ATGAAGCTTTAGGTTGGGTAACCGG
NEG_Pep1_STOP	Reverse	GTAGTCGACCAATGCCTCGAAGG
NEG_Pep2	Forward	ATGAAGCTTAGCGCGGCTACCTGGGTCGCGAGTC
NEG_Pep2	Reverse	GTAGTCGACCAATGCATCTCGCGTACGCCTGTGG
NEG_Pep2_STOP	Forward	ATGAAGCTTTAGGCGGCTTAATGGGT
NEG_Pep2_STOP	Reverse	GTAGTCGACCAATGCATCTCGCGTA

NEG_Pep3	Forward	ATGAAGCTTAGCTGTCCATTTCCGGATACCCATG
NEG_Pep3	Reverse	GTAGTCGACCAATGCACACACCCAGAAGACGTGC
NEG_Pep3_STOP	Forward	ATGAAGCTTTAGTGTCCATAACCGGATACCC
NEG_Pep3_STOP	Reverse	GTAGTCGACCAATGCACACACCC
NEG_Pep4	Forward	ATGAAGCTTAGCGTGTATATTCTTACGGTCCAGT
NEG_Pep4	Reverse	GTAGTCGACCAATGCCAGCGTGTTAGCCCGACGC
NEG_Pep4_STOP	Forward	ATGGAAGCTTTAGGTGTATTAACTTACGGTCCAGT
NEG_Pep4_STOP	Reverse	GTAGTCGACCAATGCCAGCGTG
NEG_Pep5	Forward	ATGAAGCTTAGCTCAGTTTGCATCCTTGTCCTGG
NEG_Pep5	Reverse	GTAGTCGACCAATGCCCGAGAGGGCTTGCGCTCT
NEG_Pep5_STOP	Forward	ATGAAGCTTTAGTCAGTTTAAATCCTTGTCCTGG
NEG_Pep5_STOP	Reverse	GTAGTCGACCAATGCCCGAGAGG
NEG_Pep6	Forward	ATGAAGCTTAGCAAAGTAGTTTATCGTCGCGCAG
NEG_Pep6	Reverse	GTAGTCGACCAATGCCGAGGTTACACAGACACTG
NEG_Pep6_STOP	Forward	ATGAAGCTTTAGAAAGTATAATATCGTCGCGC
NEG_Pep6_STOP	Reverse	GTAGTCGACCAATGCCGAGGTTACA
POS_Pep1	Forward	ATGAAGCTTAGCCGCGGTATTCACCTAGGTCGGA
POS_Pep1	Reverse	GTAGTCGACCAATGCGTCCAAAACCCAGTGT
POS_Pep2	Forward	ATGAAGCTTAGCTACTGGAATAGCTCTATGGCGT
POS_Pep2	Reverse	GTAGTCGACCAATGCGTCGGTATCAAACCGT
POS_Pep3	Forward	ATGAAGCTTAGCCCCGTCTCCTGGATTCACGGTG
POS_Pep3	Reverse	GTAGTCGACCAATGCATAGCTTACCCCAGGC
POS_Pep4b	Forward	ATGAAGCTTAGCGTCATGCGTCCCATATCTCG
POS_Pep4b	Reverse	TCATCGTCCTTGTAGTCGACCAATGCTTAGCG
POS_Pep6	Forward	ATGAAGCTTAGCGAAGGTGGCCGCCGAT
POS_Pep6	Reverse	CATCGTCCTTGTAGTCGACCAATGCGGTACG
POS_Pep7	Forward	ATGAAGCTTAGCGTCAAATTTGCAAGGGTTGGGGTC
POS_Pep7	Reverse	TCATCGTCCTTGTAGTCGACCAATGCACCTC
Outer_pFLAG	Forward	GCATAATTCGTGTCGCTCAA
Outer_pFLAG	Reverse	AAAAGGGAATAAGGGCGACA
